# Center point to pose: Multiple views 3D human pose estimation for multi-person

**DOI:** 10.1371/journal.pone.0274450

**Published:** 2022-09-13

**Authors:** Huan Liu, Jian Wu, Rui He

**Affiliations:** The State Key Laboratory of Automotive Simulation and Control, Jilin University, Changchun, China; Karunya Institute of Technology and Sciences, INDIA

## Abstract

3D human pose estimation has always been an important task in computer vision, especially in crowded scenes where multiple people interact with each other. There are many state-of-the-arts for object detection based on single view. However, recovering the location of people is complicated in crowded and occluded scenes due to the lack of depth information for single view, which is the lack of robustness. Multi-view Human Pose Estimation for Multi-Person became an effective approach. The previous multi-view 3D human pose estimation method can be attributed to a strategy to associate the joints of the same person from 2D pose estimation. However, the incompleteness and noise of the 2D pose are inevitable. In addition, how to associate the joints itself is challenging. To solve this issue, we propose a CTP (Center Point to Pose) network based on multi-view which directly operates in the 3D space. The 2D joint features in all cameras are projected into 3D voxel space. Our CTP network regresses the center of one person as the location, and the 3D bounding box as the activity area of one person. Then our CTP network estimates detailed 3D pose for each bounding box. Besides, our CTP network is Non-Maximum Suppression free at the stage of regressing the center of one person, which makes it more efficient and simpler. Our method outperforms competitively on several public datasets which shows the efficacy of our center point to pose network representation.

## 1. Introduction

3D human pose estimation in crowded scenes is an important component of computer vision applications such as autonomous driving [1], surveillance [2], robotics [3] and human-computer interaction [4]. When single view is used for human pose estimation, although it is widely applicated in other object-detection, the lack of depth information and occlusion directly results the incomplete pose estimation. Multiple views 3D pose estimation can compensate for the loss of joint information caused by human occlusion. However, the fusion of image information from multiple views is still a challenge.

Previous works usually propose to divide the 3D human pose estimation into three steps [5–11]: 1) Detecting human body keypoints under a single view; 2) Associating the joints of the same person across different views; 3) Recovering 3D pose in space. The whole three steps are interrelated and progressive. Despite human pose estimation under single view [12, 13] has been excellently benefit from deep-learning and the development of 2D detection methods, only rough location can be performed due to the absence and occlusion of the joints. Based on the estimated 2D pose, multiple views estimation cannot improve the accuracy when missed and false detection occur, although there is redundant information provided by multi cameras. Most research focus on steps 2) and 3) recently, which aim at matching people across different views. Some previous methods [5–8] pro-pose a 3D pictorial structure (3DPS) model that solves the question of matching by geo-metric constraints. However, the results are still unstable due to the huge state space. Then they increase the affinity matrix [9] to improve the performance. A graph model [10, 11] has also been proposed to achieve it. However, none of them do not avoid the matching task.

**Fig 1:**
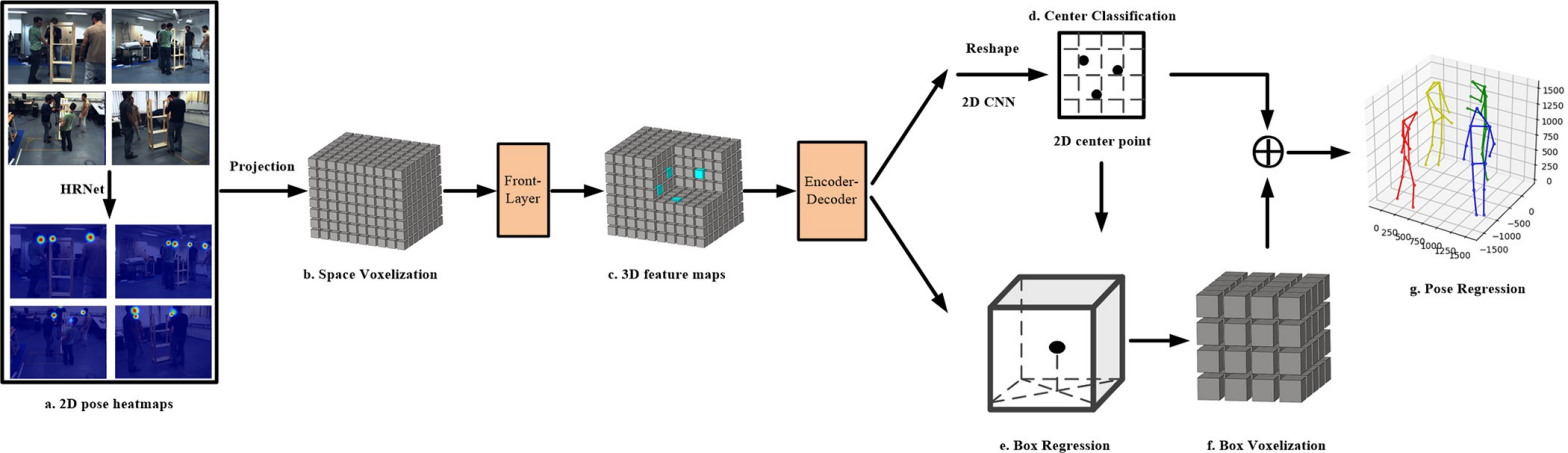
Our CTP network avoids the complex matching task. (a) We estimate 2D heatmaps from all views. (b) When all 2D keypoint heatmaps projected into 3D common space, the space is voxelized into regular grids. (c) After convolution by front-layer in backbone, we get the preliminary 3D feature maps. (d) The 3D feature maps are transformed into 2D feature maps and passed into 2D CNN network. The center of one person is generated in top view. (e)The 3D bounding box is regressed. (f) The 3D bounding box is voxelized into more detailed grids for estimating accurate 3D pose. (g) The estimation of 3D poses outputs from our network.

To avoid the challenge of matching task and reducing the costs, we propose a 3D pose estimator which operates in space with multiple views projection information. In-spired by the success of CenterNet3D [14], We propose our CTP network, as shown in Fig 1, to predict the center area of one person and the 3D bounding box, and then regress the detailed pose of each person. Firstly, it estimates 2D heatmaps from each view to encode per-pixel likelihood of all joints, all of them are projected into one 3D common space. This approach avoids assigning joints to different instances. Secondly, the 3D space is voxelized into regular grids and each grid represents an anchor. The regular grids pass through the 3D CNN network of our backbone to regress a confidence score, all anchors form a 3D heatmap. Then the 3D feature maps are transformed into 2D feature maps and passed into 2D CNN network by collapsing in Z axis. The center of one person is generated in top view. Then a regressed fixed orientation and size cube big enough represents a 3D bounding box of one person to describe the activity area. The bottom of the 3D bounding box shares the same center with the regressed center of one person. We take half the height of the 3D bounding box as the Z coordinate of the center point of one person. And then, the anchor where the center point with spatial coordinates locates in represents one person. We will determine the detailed locations of joints after regressing the bounding box. Lastly, the bounding box is taken as a new 3D common space to estimate complete pose. Due to the 3D bounding box is voxelized detailed enough, each joint is regressed by the 3D CNN network in our backbone network. In our method, each stage of our CTP work shares the same joint heatmaps. In the 3D space, the number of human joints is limited and sparse. In order to deal with this sparse data, we redesign a concise backbone network, which has high resolution output to deal with sparse information.

The main contributions of our method can be summarized as follows:

We propose a novel CTP network for sparse keypoints of human body which is not as rich as the dense Lidar point cloud data, our work directly handle the projected 2D pose information in 3D space without associating the joints of the same person from 2D pose estimation. Each person is represented by a center area and bounding box and the detailed pose will be regressed in 3D bounding box.

We redesign a concise backbone network to adapt to our voxelized data.

The proposed approach outperforms competitively on three public datasets including Campus, Shelf and CMU Panoptic.

## 2. Related work

In this section, we briefly review previous 3D pose estimation works from singe person to multi-person. In these studies, we can find that the influence of occlusion on 3D pose estimation. We briefly illustrate the previous works, we take it whether they employ multiple views as the standard.

### 2.1. Single person 3D pose estimation

Single view 3D pose estimation develop rapidly due to the advancement of deep learning. In some recent researches, a coarse-to-fine prediction method was proposed [15–17] by estimating 3D human pose in a volumetric representation. In order to unify the heatmap representation and joints regression, Integral pose [18] proposed a method that the non-differentiable argmax can be replaced with integral operation. No matter how excellent of these methods in single view, occlusion is still an inevitable issue, especially for multi-person, one pose associated multiple keypoints of different person directly results the inaccurate count on persons. In order to reduce ambiguities, previous works [19–21] optimized the low-dimensional parameters of the representation to minimize the discrepancy between its projection and the 2D pose. The improvement of deep neural networks aims to deal with the ambiguity issue, such as regressing 3D pose from 2D joint locations directly [22, 23], projecting 2D features or 2D keypoint heatmaps to 3D space to estimate 3D locations, which is more effective than triangulation. But in multi-person scenes, associating joints to persons is still a necessary part.

### 2.2. Multi-person 3D pose estimation

Although multiple views 3D pose estimation [5, 6, 24–26] can compensate for the occlusion in single view [27], bottlenecks still exist in these methods. Multiple views pose estimation provides additional information, triangulation is a simple geometric method. However, it depends on the performance of 2D pose estimation, when expanded to multi-person scenes, a joint in one view should lie on the epipolar line associated with its correspondence in another view. Whether adding appearance features or reducing the noise of triangulation [9, 28] by either top-down [29, 30] or bottom-up [12, 31, 32], they need to associate both joints and 2D pose of the same person. The graph model [10, 11] were applicated in multi-person scenes. The complexity of this method increases the cost, especially when the number of people is assumed to be known, which itself should be another detection process. Our work avoids the association task and the local optima unlike graph model [10] and Bipartite graph [11].

## 3. Technical approach

Inspired by the CenterNet3D [14], our CTP network expands lidar 3D object detection work to 3D pose estimation. The 3D space is voxelized into grids, each voxel represents an anchor, of which likelihood is predicted a proposal of one person. We take the center of the anchor as the center of one person. Our work is same as CenterNet3D without NMS. Then we employ a 3D bounding box to describe the activity area of one person.

The proposed CTP network consists CTP head and pose regression module (PRM). The CTP head has three modules for voxelized coding, center classification and box regression. In CTP head, the front-layer of our backbone extracts the preliminary 3D feature maps and 3D feature maps are transformed into 2D feature maps, then the 2D CNN in encoder-decoder module of our backbone generate a rough center point of one person. Then a 3D bounding box is regressed based on the center point. In PRM, the 3D bounding box is taken as a new common space, our 3D CNN network in encoder-decoder module slides on new common space and the output of the network will be the 3D pose. The process of CTP network is shown in Fig 1. In next section, we only illustrate the process of our work, and detailed experiment parameters will show in section IV.

### 3.1. CTP head

#### 3.1.1 Voxelized coding

When the 2D heatmaps are projected into the 3D common space, the space of size *R*_*x*_×*R*_*y*_×*R*_*z*_ are voxelized into regular grids *I*∈*R*^*L*×*W*×*H*^ along *X*×*Y*×*Z* axes respectively. *v*_*x*_, *v*_*y*_ and *v*_*z*_ denote the length of voxels at each direction. The number of voxels in each direction can be computed as *L* = *R*_*x*_/*v*_*x*_, *H* = *R*_*y*_/*v*_*y*_ and *W* = *R*_*z*_/*v*_*z*_ Considering the varied scale of the persons, it first estimates 2D heatmaps from all views by HRNet [33], of which the high-resolution output is much more adaptable. We refer to [34] to voxelize the 3D space and compute average valves for each anchor simply, because we estimate the location of one person roughly. Denote the 2D heatmap of view *V* as *H*_*V*_∈*R*^*L*×*W*×*H*^ which is projected at the location *P*_*V*_^*L*×*W*×*H*^. Then we compute the average heatmap for each anchor as *H*^*m*^. Two main steps of our CTP head to locate one person are center classification and box regression. The details of these steps will be described below. The *H*^*m*^ shows as follows:

$$H^{m}=(1/V)\sum_{v=1}^{V}H_{V}$$ (1)

#### 3.1.2 Center classification

To generate the center of one person, the regular grids pass through the 3D CNN front-layer of our backbone to regress a confidence score, all anchors form a 3D heatmap $H_{DT}^{V}$. Then the 3D feature maps are transformed into 2D feature maps and passed into 2D CNN encoder-decoder module by collapsing in Z axis. The center of one person is generated on the common space ground in top view. The CenterNet3D regress the offset to recover the 2D discretization when regressing the 3D bounding box, in other words, the center of the 3D bounding box in CenterNet3D is the center of one object. In our work, the center of one person may locate at any anchor in 3D space, of which the center represents the center of one person. Unlike CenterNet3D, we do not need compute the offset of the center. Due to the flexibility of human pose, the activity area of one person is changing in real time and the center may locate on or out of the human body. In addition, the anchor is sized so small compared with the scale of one person to locate it that the offset can be ignored. We finally need to regress the human pose and it is enough to know where one person is located. Therefore, the offset is not serious for the center location of one person. On the contrary, the complex calculation steps are reduced. We only need to take half the height of the 3D bounding box as the Z coordinate of the center point of one person in space. And then, the center point has spatial coordinates. The anchor where the center point locates in represents one person. We will illustrate how to get the 3D bounding box in next section.

The center classification generates center heatmaps with several center heatmaps and each heatmap corresponds to one category. When the 3D feature maps are feed into 2D CNN network, we denote $H_{c}\in [0,1{]}^{\frac{L}{R}\times \frac{W}{R}\times c}$ be the center heatmaps, where R is the downsampling stride and *c* is the number or center point. The output prediction *H*_*c*_ is downsampled by the factor *R*. A prediction *H*_*c*_ = 1 corresponds to a detected person and *H*_*c*_ = 0 is none. Unlike CenterNet3D, we do not generate ground truth center heatmaps for training, our center point is more like a datum point for estimating human pose. The ground truth center in CenterNer3D is used to calculate the offset and our center point is used to train CTP head module to evaluate the confidence that a person exists in a spatial location.

#### 3.1.3 Box regression

After confirming the location of one person by generating the center, we need a 3D bounding box to envelope the activity area of one person as the common space of PRM. The 3D bounding box in PRM will also be voxelized into grids, so we simplify our work by employing a fixed orientation and size cube as the 3D bounding box. The bottom of the 3D bounding box shares the same center with the center point from center classification module. The center of one person adopts half the height of the 3D bounding box as the Z coordinate. We have illustrated the reason of neglecting the offset when generate the center, so the same reason for the 3D bounding box scale selection, a person’s active area is constantly changing, and we also take the convenience of voxelizing the 3D bounding box in the PRM into consider.

To train a robust CTP head, we compute a loss by minimizing the distance between the center point to other keypoints. In other word, we should compute the loss between ground truth heatmap $H_{GT}^{V}$ of the anchor and the detected 3D heatmap $H_{GT}^{V}$. We train the CTP head by the loss as follows:

$$L_{CTP-Head}=min\sum_{x=1}^{L}\sum_{y=1}^{W}\sum_{z=1}^{H}\left|H_{DT}^{V}-H_{GT}^{V}\right|$$ (2)

### 3.2. Pose regression module

In this section, the 3D bounding box is taken as a new 3D common space, the box of size *R*_*x*_′×*R*_*y*_′×*R*_*z*_′ are voxelized into regular grids *I*′∈*R*^*L*′×*W*′×*H*′^ along *X*′×*Y*′×*Z*′ axes respectively. *v*_*x*_′, *v*_*y*_′ and *v*_*z*_′ denote the length of voxels at each direction. The number of voxels in each direction can be computed as *L*′ = *R*_*x*_′/*v*_*x*_′, *W*′ = *R*_*y*_′/*v*_*y*_′ and *H*′ = R_*z*_′/*v*_*z*_′. The grids are much more detailed to generate accurate 3D pose. The approach of the voxel method is the same as CTP head except the anchor scale. The 3D CNN network in center classification module is rough to generate the 3D feature maps for multiple person. So we generate more accurate 3D heatmaps *H*_*p*_ to estimate the joints with a new 3D CNN structure in encoder-decoder module of our backbone, and the joints can be described by the center of mass of *H*_*p*_. We denote the 3D joint center as *C*_*p*_ [17]. The formula is shown as:

$$C_{p}=\sum_{x=1}^{L^{\prime }}\sum_{y=1}^{W^{\prime }}\sum_{z=1}^{H^{\prime }}\left(x,y,z\right)\times H_{p}(x,y,z)$$ (3)

We employ the L1 loss to train pose regression module. In order to decrease the cost, different joints share the same weigh. The loss between estimated K joints *H*_*k*_ and ground truth joints $H_{k}^{gt}$ is shown as:

$$L_{PRM}=\sum_{k=1}^{K}\left|H_{k}^{gt}-H_{k}\right|$$ (4)

### 3.3. Backbone network

The common space and the bounding box are voxelized after the 2D heatmaps projected in the common space. We propose a new backbone for CTP head and PRM. The number of human keypoints is not as dense as lidar point cloud data. However, V2V-PoseNet [35] is effective in 3D keypoins estimation. We refer to their design of its basic convolution module in our backbone.

**Fig 2:**
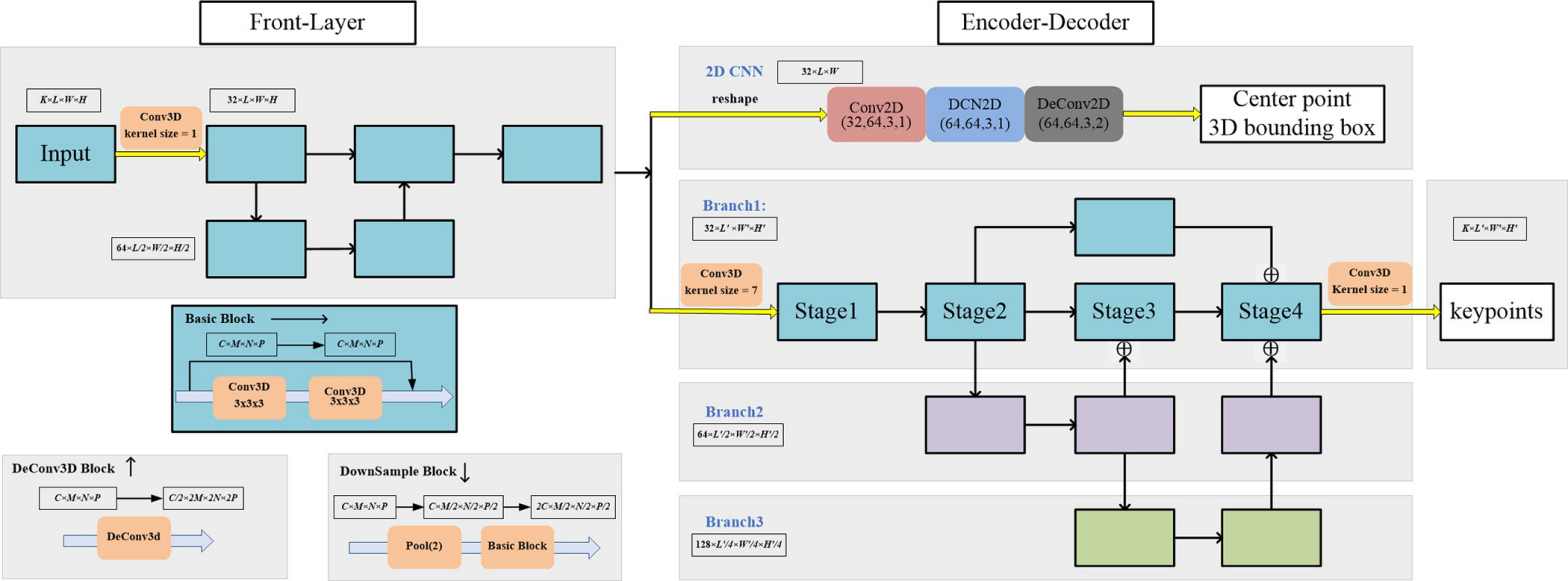
The structure of our backbone. The inputs are the voxel common space with all projected 2D heatmaps. The CTP head outputs the center of people and the bounding box, then the 3D pose comes from PRM. *C*, *M*, *N*, and *P* represent the input parameters of convolution block.

Front-Layer: This layer is used to extract 3D preliminary features. 3D residual block is adopted as the basic block. The input features are downsampled and upsampled by the basic block and then added in series, we propose a simple network. The detailed structure of front-layer is shown in Fig 2.

Encoder-Decoder Module: We just use the simple 2D convolution block like CenterNet3D [14] to get the center point, including 2D convolution, deformable convolution and transposed convolution, because we get only a rough center location. To get detailed 3D human pose, we design a new 3D convolutional network for the PRM. Inspired by HRNet [33], high resolution output can increase the detection accuracy. The success of HRNet is due to the fusion of high and low resolution at every stage and branch, as well as high resolution as the main branch. We extend it to 3D convolution. Our backbone uses 3D residual block as the basic block but our backbone is simplified to complete it. We fuse all modules at the fuse layer in stage4 of branch1. The process of CTP head and PRM is similar, they share the same heatmaps only different inputs and outputs. For example, we should replace the *L*×*W*×*H* as *L*′×*W*′×*H*′. The structure of the backbone network shows in Fig 2.

## 4. Empirical evaluation

### 4.1. Datasets

We select three public datasets to evaluate our work. The participants involved in the datasets are all available in the public dataset and can be used for research.

Campus: It is a dataset consisting of three people in an outdoor environment. There are three people interacting with each other under three calibrated cameras. We follow the previous work [5–7, 9] and divide the datasets into training and testing subsets with the same percentage. The evaluation metric is 3D Percentage of Correct Parts (PCP).

Shelf [5]: Compared with Campus, this dataset adds calibrated cameras to five. Four people disassemble a shelf indoors, where people occlusion make it complex. The evaluation metric is also 3D PCP.

CMU Panoptic [8]: This dataset is also captured indoors, but the large number of cameras reaches to hundred in the studio. It contains more people than Shelf and Campus datasets. We only randomly select five cameras large enough to capture panoramas for training qualitatively evaluate our network on the CMU Panoptic dataset as same as it in [9]. We select “160226_haggling1”, “160422_haggling1”, “170915_office1”, “160906_ian1” as training set and “160224_haggling1”, “160422ultimatum1” for testing set. All sets contain multiple people and the number of people various. The evaluation metric Mean Per Joint Position Error (MPJPE).

When we visual the results of 2D and 3D pose estimation on these three datasets, all 2D poses in the results are projected from estimated 3D poses, instead of the 2D pose from HRNet. In addition, we also show the occluded joints on the shelf dataset, where some joints cannot be detected by some cameras.

### 4.2. Training parameters

#### 4.2.1 3D bounding box scale

The space scale on public datasets is *R*_*x*_×*R*_*y*_×*R*_*z*_ = 8*m*×8*m*×8*m*. In terms of people scale, the size in each orientation of one person extends within 2*m*. A *R*_*x*_′×*R*_*y*_′×*R*_*z*_′ = 2*m*×2*m*×2*m* area is enough for various 3D pose because the arms span of one person is roughly the same as his height and generally within *R*_*x*_′×*R*_*y*_′×*R*_*z*_′ = 2*m*×2*m*×2*m*. So that the bounding box is a 2*m* cube, which is also the voxel space inputs of the PRM.

#### 4.2.2 Voxel group

We should select a moderate anchor size to balance between speed and accuracy for CTP head, and the number of anchors directly affects the computational complexity of convolutional networks. In addition, we do not compute the offset of the center point, the anchor should not be set too large. The anchor is set as a cube with the size of *v*_*x*_ = *v*_*y*_ = *v*_*z*_ = 0.1*m*. The common space is voxelized *L*×*W*×*H* = 80×80×20 grids along each axis. This approach is also verified reasonable in our ablation analysis. The space in PRM voxelized into *L*′×*W*′×*H*′ = 64×64×64 grids along each axis is enough to estimate accurate 3D pose, the anchor is set as a cube with the size of *v*_*x*_′ = *v*_*y*_′ = *v*_*z*_′ = 0.03*m* approximately.

#### 4.2.3 Training strategy

Our work avoids the match of the joints and people, but 2D pose still needed. We pretrain 2D pose estimation with HRNet. 50 epochs for jointly training the CTP head and PRM. We select the similar method [33] for learning rate, the initial learning rate is set to be 1e-3, and then dropped 10^×^ at 20 and 40 epochs. The network is trained by ADAM [36] optimizer. In particular, in the first 20 epochs, we can set the 3D bounding box space anchor scale as 0.1*m* to increase the training speed, which was found in our ablation experiments.

### 4.3. Ablation analysis

In this section, we run an ablation analysis to justify the design of our training parameters. The Shelf dataset is used for evaluation, we select our initial work as the best estimated 3D pose to compare the deviation with each other, because the incorrect 3D pose itself may be influenced by many inestimable factors. We choose the 2D pose from camera3 and camera5 and 3D pose for comparative experiments.

#### 4.3.1 Common space voxelization

**Fig 3:**
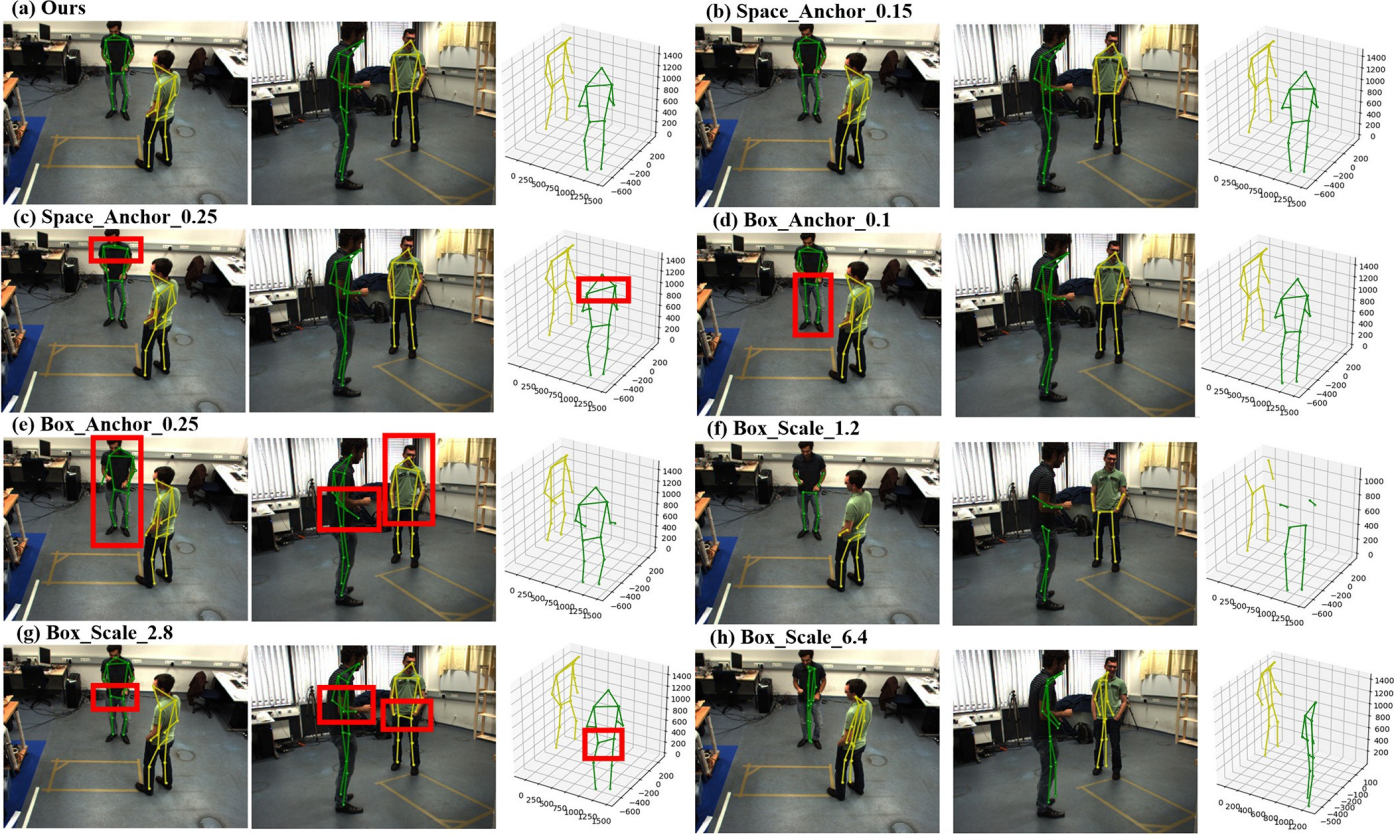
The results of ablation analysis experiment. (a) shows our best result and it is chosen as the benchmark for comparison. (b) and (c) shows the visual result for changing the 3D common space voxel anchor scale as 0.15*m* and 0.25*m*. (d) and (e) shows the visual result when the 3D bounding box voxel anchor scale is changed as 0.1*m* and 0.25*m*. In (f), (g) and (h), the 3D bounding box scale is changed as 1*m*, 2.8*m* and 6.4*m* for ablation analysis. In order to speed up calculations, the 3D bounding box voxel anchor scale is set to 0.1*m* and compared with (d).

In order to determine the effect of common space voxel scale on generating the center point of one person, we should change the voxel scale to verify it. In our best result, the size of the anchor we choose is based on the actual joint size of most people. For example, if one anchor envelope all joints of all people, we cannot distinguish multiple people. So the common space voxel anchor scale should not be too large. The anchor scale is serious for regressing the center point of one person. We select three anchor size for space voxel anchor, including 0.1*m* (our work), 0.15*m* (Half the body width of most people), and 0.25*m* (The larger anchor scale, the larger center offset may exist compared with the ground truth center of one person). We can see that when the anchor size increase from 0.1*m* to 0.15*m* and 0.25*m*, the PCP decrease by 0.6% and 5.1% compared with our best result in Table 1B and 1C. We can know the larger anchor scale the larger center offset of the location prediction should occur. Therefore, the 3D bounding box position placed base on the center location of one person and the anchor position after the bounding box voxelizated will change a lot. The error location of one person will influence the position of estimated joints in the anchor of the 3D bounding box and the recovery of the 3D pose. For example, when the 3D bounding box anchor scale is equal in any experiment, due to a large center point offset of one person, the location of the 3D bounding box should change. A joint belongs to one anchor but will be located in another anchor in the 3D bounding box. In Fig 3B, when the anchor scale increase from 0.1*m* to 0.15*m*, the estimated joints location change little almost we cannot distinguish by our eyes, only the PCP decrease by 0.62%, small common space voxel anchor scale has an insignificant influence. But when increasing to 0.25*m*, in Fig 3C, the shoulder location for one actor has a large offset. The result shows that the common space anchor scale has an obvious impact on PCP, but not large for regressing complete 3D pose only with a large offset of the joints. Therefore, we can appropriately adjust the anchor scale to meet our needs in practical applications. For example, we only focus on analyzing human movements without considering precise joint positions, which can reduce the computational costs.

**Table 1:** The PCP result of ablation about our CTP network. The number in parameters name represent the anchor scale or box scale.

Parameters	Space voxel	Box voxel	Box scale	Actor1	Actor2	Actor3	PCP
**(a) Ours**	0.1	0.03	2	**99.3**	**94.3**	**97.7**	**97.1**
**(b) Space_Anchor_0.15**	0.15	0.03	2	98.9	93.0	97.5	96.5
**(c) Space_Anchor_0.25**	0.25	0.03	2	94.4	85.1	96.4	92.0
**(d) Box_Anchor_0.1**	0.1	0.1	2	**98.6**	**95.4**	**97.5**	**97.2**
**(e) Box_Anchor_0.25**	0.1	0.25	2	93.4	83.5	89.9	88.9
**(f) Box_Scale_1.2**	0.1	0.1	1	68.6	59.5	68.0	65.4
**(h) Box_Scale_2.8**	0.1	0.1	2.8	97.2	91.4	96.9	95.2
**(g) Box_Scale_6.4**	0.1	0.1	6.4	60.6	58.4	57.8	58.9

We do not select a smaller anchor size compared with 0.1*m* due to the slightly improvement but more costs, and 0.1*m* is enough to envelope any joint for one person, also enough to get an accurate 3D pose and PCP. We just need to get preliminary 3D feature maps in voxel space. In addition, the balance between speed and accuracy has been proven reasonable by VoxelPose [37].

#### 4.3.2 3D bounding box voxeliztaion

Although increasing common space voxel scale will reduce PCP, we can compensate for this error by detailed voxelization 3D bounding box. To verify the assume, we set the voxel anchor scale of 3D bounding box to 0.1*m* and 0.25*m*. In Table 1D and 1E, the average PCP from 88.9% increase to 97.2%. We consider it with differential view, when the voxel anchor scale is as small as the scale of the joint point, then the joint point will not fall into any wrong anchor. It also indirectly shows that the accuracy of our method depends on the complementarity of space voxel and 3D bounding box voxel scale. In addition, in this part of ablation experiment, we found that the network training speed increases as the 3D bounding box anchor scale increase, which also proves that the scale of 0.1*m* is reasonable in VoxelPose [37]. Although the PCP result in Table 1D surpasses (a), the PCP for each actor is unsatisfactory. We choose (a) in Table 1 as our best method. Furthermore, it also proves that a poor effect for increasing average PCP by the more detailed voxelization, so we do not select 3D bounding box voxel scale less than 0.03*m*.

When the 3D bounding box voxel anchor scale increases to 0.1*m*, the pose around waist has a small offset compared with our best result in Fig 3A. When increasing to 0.25*m*, in Fig 3E, besides the wrist of one actor was located incorrect, the larger anchor scale, the larger offset in all the pose about one actor. The voxel scale directly affects the quality of 3D feature maps obtained by our backbone, and poor-quality convolution calculation cannot obtain accurate 3D feature maps position.

We prove the assume is correct only base on the 3D bounding box is a cube with the scale of 2*m*, which means that one 3D bounding box only envelops as few people as possible. We also need to explore the effect of the 3D bounding box scale on regressing 3D pose. In other words, since we set 3D bounding boxes as fixed orientation and size cubes instead of regressing accurate boxes, we need to explore the difference between our method and the top-down method.

#### 4.3.3 3D bounding box scale

We select three scale of the bounding box for PRM, including 1.2*m* (The human body cannot be enveloped), 2*m* (Small enough to envelop most human body), and 2.8*m* (Excessive area to envelop most human body). In order to speed up calculations and there is only a small offset in visual for 3D poses although the PCP is not the best, we take the result in Table 1D where the 3D bounding box voxel anchor scale is set to 0.1*m*, as another comparison experiment, it is also worth to be compared. The comparison shows that our work is similar to traditional top-down methods only we save the computational cost of regressing the bounding box, the accuracy of the 3D bounding box size influences the pose estimation.

The 3D bounding box is so small that some joints may not associate with any other joint to generate a limb, which results a low PCP in Table 1F. In Fig 3G, we can see that the limb outside the box cannot be estimated due to the 3D bounding box starts from the ground. However, the 3D bounding box scale is not as better as larger although it large enough. In section 3.1.3, we compute a loss by minimizing the distance between the center point to other keypoints in Eq (1), all the joints in the 3D bounding box from any actor should be computed in the loss. A large 3D bounding box may contain multiple human keypoints, but as long as the anchor in 3D bounding box is detailed enough, it may not cause one joint associated to multiple people belong to the same anchor. However, due to the uncertainty of the center point of one person we determined, the joints of any other people in the 3D bounding box may also be closer to the center point when calculating the loss. One box with a suitable scale contains a small number of joints from other people and these joints are only calculated in the loss calculation with a little weight. In Fig 3G, the limbs near the waist and the wrist are estimated with a large offset. In order to observe obviously, we set it as a cube with the scale of 6.4*m*. In Fig 3H, the estimated 3D poses are twisted. Once these loss weights cannot be ignored, the location of the joints and pose should be away from the ground truth. To introduce the weights to joints loss function calculation may be our future research work in multiple views 3D human pose estimation for multi-person.

### 4.4. Comparison with previous works

**Fig 4:**
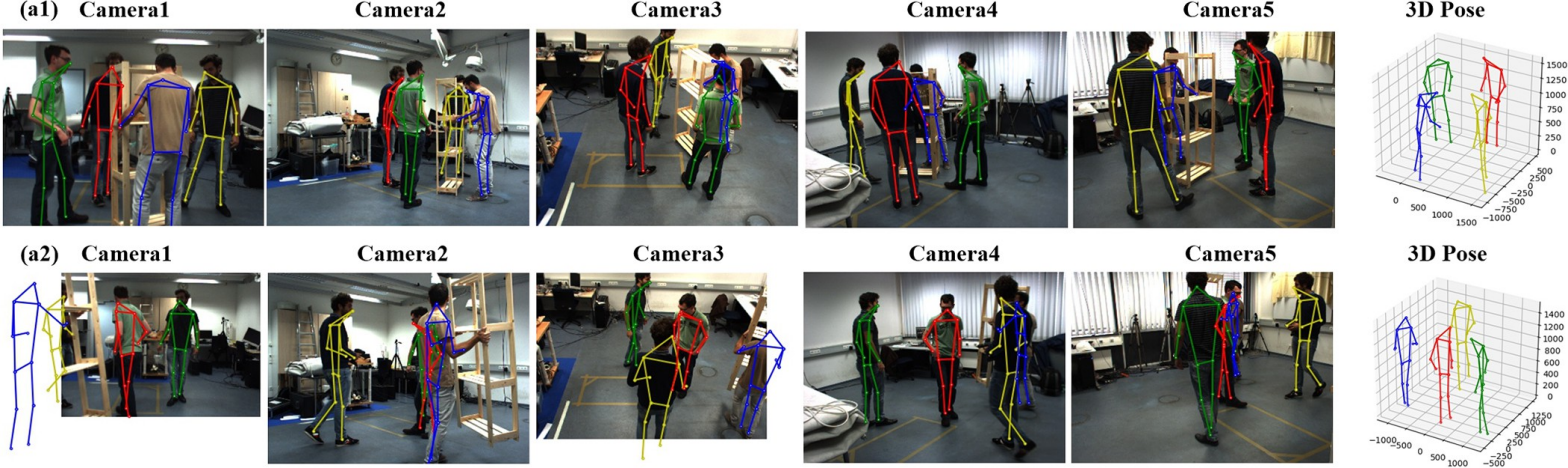
The visual results on shelf dataset. (a1) is the regular show on shelf dataset. (a2) shows the projected 2D pose from 3D pose and the 2D poses are not the estimated poses from HRNet. The result in (a2) shows that the multiple views 3D pose estimated can compensate for invisible pose information.

**Fig 5:**
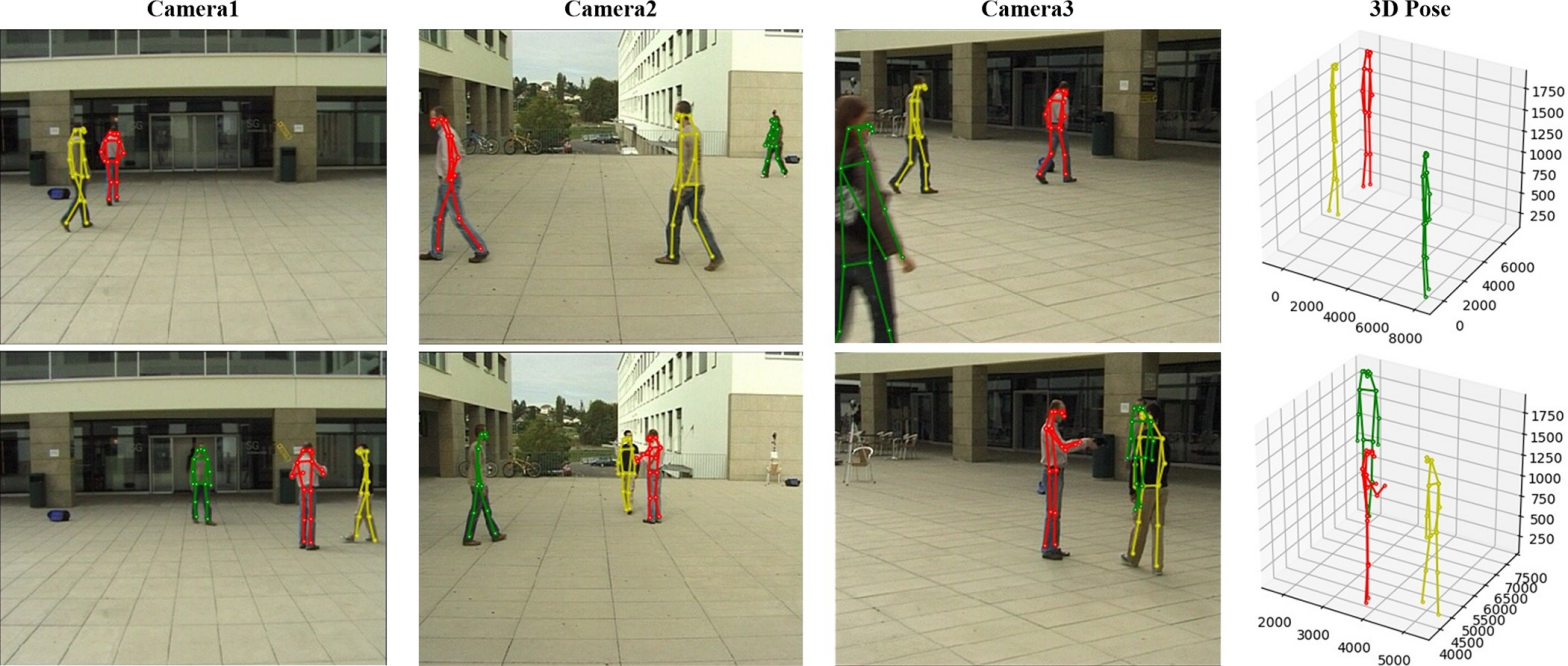
The result shows the 3D pose estimation on Campus dataset. There are only three people with small scale, the complete 3D poses are also estimated, which proves that our backbone is effective.

We compare our work with some previous methods. The results on Shelf and Campus datasets show in Table 2, Figs 4 and 5. Different from previous matching methods, our CTP network inspired by CenterNet3D and HRNet and directly operates in 3D common space. Our method proposes a novel and more simple approach to recover the 3D pose and the experiment proves that our CTP network outperforms competitively on these datasets. Our method increases the detection accuracy of Actor2 like [38]. In fact, we can see that our results on Actor2 in Table 1D is 95.4% and better than [38], but we should consider the results of every Actor comprehensively, we also explained the reasons for which set of results to choose as the best in section 4.3.2. Although there are only three persons in Campus, the result shows that our backbone is adaptable to the people with small scale in Campus dataset.

**Table 2:** Quantitative comparison of campus and shelf datasets with PCP. Results for other methods are taken from their respective papers.

**Shelf**	**Actor1**	**Actor2**	**Actor3**	**Average**
**Belagianniset al. [5]**	66.1	65.0	83.2	71.4
**Belagianniset al. [24]**	75.0	67.0	86.0	76.0
**Belagianniset al. [6]**	75.3	69.7	87.6	77.5
**Ershadi-Nasabet al. [7]**	93.3	75.9	94.8	88.0
**Donget al. [9]**	98.8	94.1	97.8	96.9
**VoxelPose [37]**	**99.3**	94.1	97.6	97.0
**MvP [38]**	**99.3**	**95.1**	**97.8**	**97.4**
**Ours**	**99.3**	94.3	97.7	97.1
**Campus**	**Actor1**	**Actor2**	**Actor3**	**Average**
**Belagianniset al. [5]**	82.0	72.4	73.7	75.8
**Belagianniset al. [24]**	83.0	73.0	78.0	78.0
**Belagianniset al. [6]**	93.5	75.7	84.4	84.5
**Ershadi-Nasabet al. [7]**	94.2	92.9	84.6	90.6
**Donget al. [9]**	97.6	93.3	98.0	96.3
**VoxelPose [37]**	97.6	93.8	**98.8**	**96.7**
**MvP [38]**	**98.2**	**94.1**	97.4	96.6
**Ours**	97.8	93.6	98.3	96.6

**Fig 6:**
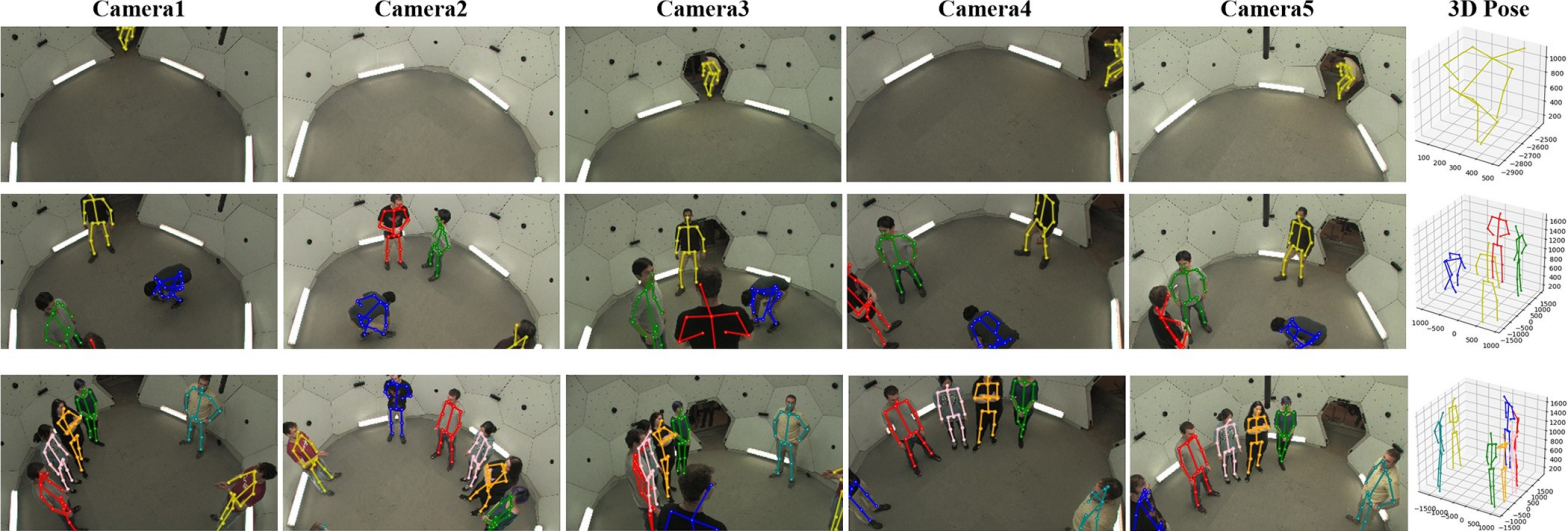
The multiple people visual result on CMU Panoptic dataset. We select some datasets with different numbers of people to evaluate our method. Our method is also robust in multi-person scenes with more than 5 people.

In addition, previous works only use CMU Panoptic dataset for qualitative evaluations or single-person pose detection discarding multi-person scenes [9, 11]. We compare it with VoxelPose [37] and MvP [38] for evaluations. Table 3 further shows that our method decreases the error by ~0.6mm on Panoptic dataset compared with VoxelPose. In some aspects, our results are better than the recent MvP method such as AP_50_, AP_100_ and AP_150_. The result in CMU Panoptic dataset shows that our work successfully detected a small scale person at the door in Fig 6, which proves the effectiveness of our backbone network.

**Table 3:** Comparison with [37] on CMU Panoptic dataset under 5 views.

CMU	Views	AP_25_	AP_50_	AP_100_	AP_150_	MPJPE
**VoxelPose [37]**	5	84.0	96.4	97.5	97.8	17.8mm
**MvP [38]**	5	**92.3**	96.6	97.5	97.7	**15.8mm**
**Ours**	5	86.5	**97.2**	**98.9**	**99.5**	17.1mm

### 4.5. Qualitative evaluation

Our network avoids the challenging task to associating the joints of the same person, which also avoids an extra computational cost. Our CTP network voxelizes the 3D common space when the 2D heatmaps projected into it. Then all steps are operated into this common space and our work is adaptative to the sparse space. The results show that our approach outperforms competitively on public datasets even surpass previous work in several aspects. Figs 4–6 shows our visual results on three datasets. The 2D poses in the figures are all projected from the estimated 3D pose, not the 2D pose estimated by HRNet. Fig 4 (a2) shows the 2D poses in the blind spot of all cameras. In particular, we test on multi-person scenes with more than four people in CMU Panoptic dataset to verify its robustness. The result in Fig 6 shows that we can predict their 2D and 3D pose precisely.

## 5. Conclusion

In this paper, we propose a novel approach to estimate 3D human pose for multi-person on multiple views. Compared with the previous methods, our key object is to avoid associating the joints of the same person across different views, which is challenging and costly. Inspired by CenterNet3D and HRNet, we innovatively expand it to 3D pose estimation and propose a CTP (Center Point to Pose) network to fuse all 2D projected heatmaps to estimate 3D pose in the 3D space. The experimental results on public datasets prove the effectiveness and competitiveness of our method.

## Supporting information

S1 File Datasets description. The details of experimental datasets are described in the separated S1 File.
(DOCX)
