# Supplementary material for: Center point to pose: Multiple views 3D human pose estimation for multi-person
Source: PLoS One. 2022 Sep 13;17(9):e0274450. doi: 10.1371/journal.pone.0274450 (PMC9469997; doi:10.1371/journal.pone.0274450)
Supplement: S1 File — The details of experimental datasets are described in the separated S1 File. (DOCX) [file pone.0274450.s001.docx]

Dataset Description

We appreciate your suggestion.

The Campus [1-3, 5] dataset, Shelf dataset [1] and CMU Panoptic dataset [4] are applied to train and test our proposed method.

**Campus and Shelf datasets**

Campus and Shelf datasets are challenging 3D human pose datasets for multiple human 3D pose estimation. Campus is a dataset consisting of three people in an outdoor environment. There are three people interacting with each other under three calibrated cameras. Previous work [1-3, 5] divide the datasets into training and testing subsets with a certain percentage. The evaluation metric is 3D Percentage of Correct Parts (PCP). Some Campus dataset example images and 3D ground truth joints are shown in S1 Fig.

Compared with Campus dataset, Shelf [1] dataset adds calibrated cameras to five. Four people disassemble a shelf indoors, where people occlusion make it complex. The evaluation metric is also 3D PCP. Some Shelf dataset example images and 3D ground truth joints are shown in S2 Fig. The datasets could be downloaded from online publicly as follow.

Campus and Shelf datasets:

<http://campar.in.tum.de/Chair/MultiHumanPose>


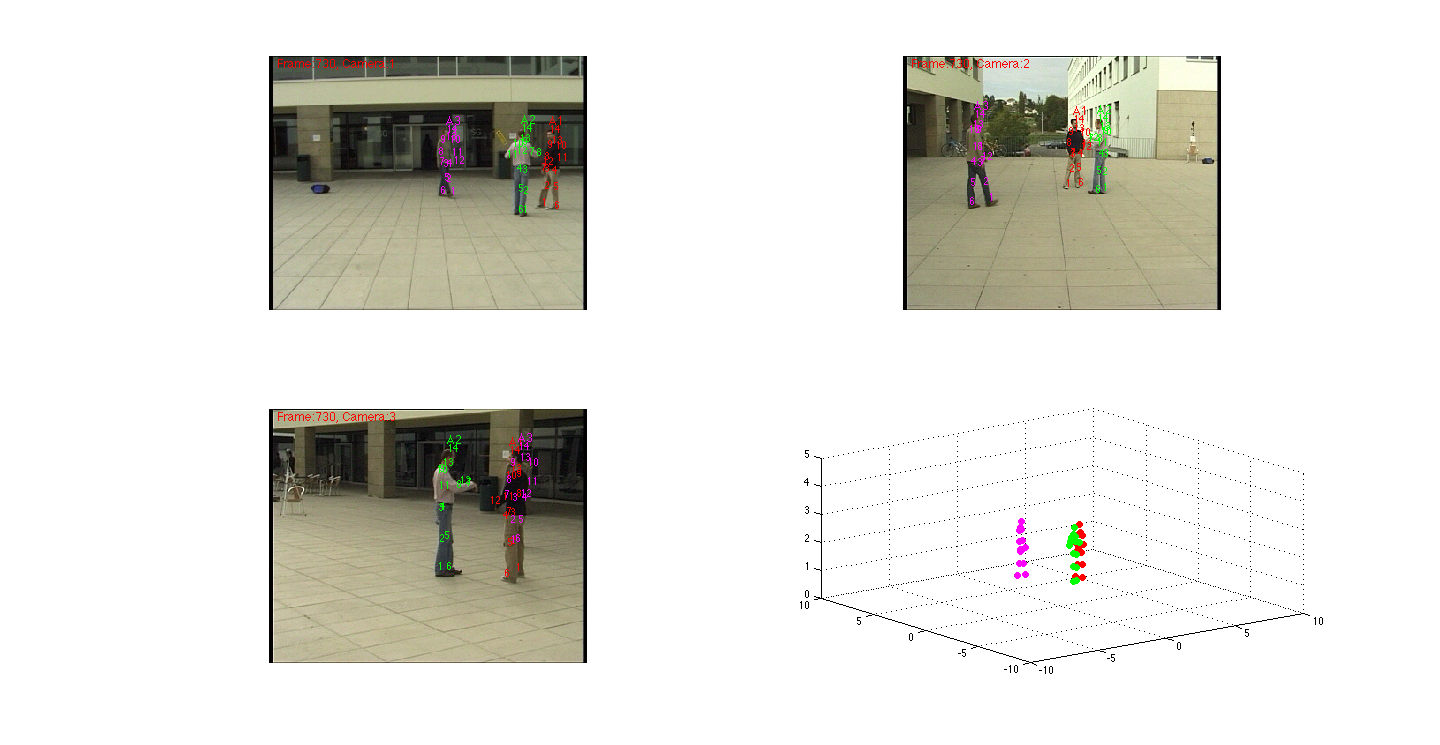


**S1 Fig.** Some example images of Campus dataset and 3D ground truth joints


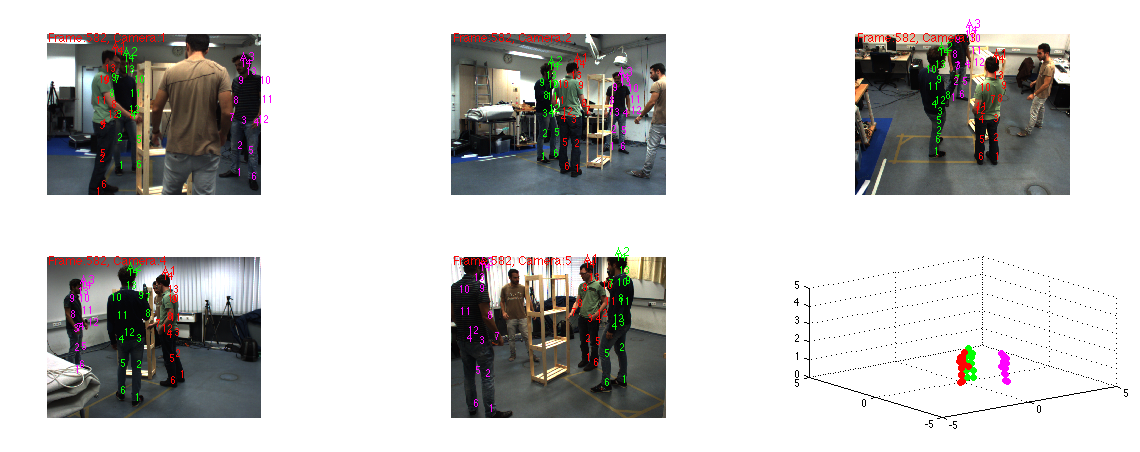


**S2 Fig.** Some example images of Shelf dataset and 3D ground truth joints

**CMU Panoptic dataset**

CMU Panoptic dataset [4] is also captured indoors, but the large number of cameras reaches to hundred in the studio. It contains much more people than Shelf and Campus datasets, as well as much more scenes. Currently, 65 sequences (5.5 hours) and 1.5 millions of 3D skeletons are available. The massively Multiview System contains 480 VGA camera views, 30+ HD views and 10 RGB-D sensors. It is Hardware-based sync and all cameras are calibrated. The evaluation metric Mean Per Joint Position Error (MPJPE).

CMU Panoptic dataset keeps upgrading their system. Currently the system has the following hardware setup: 1). 480 VGA cameras, 640 x 480 resolution, 25 fps, synchronized among themselves using a hardware clock; 2). 31 HD cameras, 1920 x 1080 resolution, 30 fps, synchronized among themselves using a hardware clock, timing aligned with VGA cameras; 3). 10 Kinect Ⅱ Sensors. 1920 x 1080 (RGB), 512 x 424 (depth), 30 fps, timing aligned among themselves and other sensors; 4). 5 DLP Projectors. synchronized with HD cameras.

CMU Panoptic Studio dataset is shared only for research purposes, and this cannot be used for any commercial purposes. The dataset or its modified version cannot be redistributed without permission from dataset organizers. Some CMU Panoptic dataset example images and 3D ground truth joints are shown in S3 Fig. The datasets could be downloaded from online publicly as follow.

CMU Panoptic dataset

<http://domedb.perception.cs.cmu.edu/>


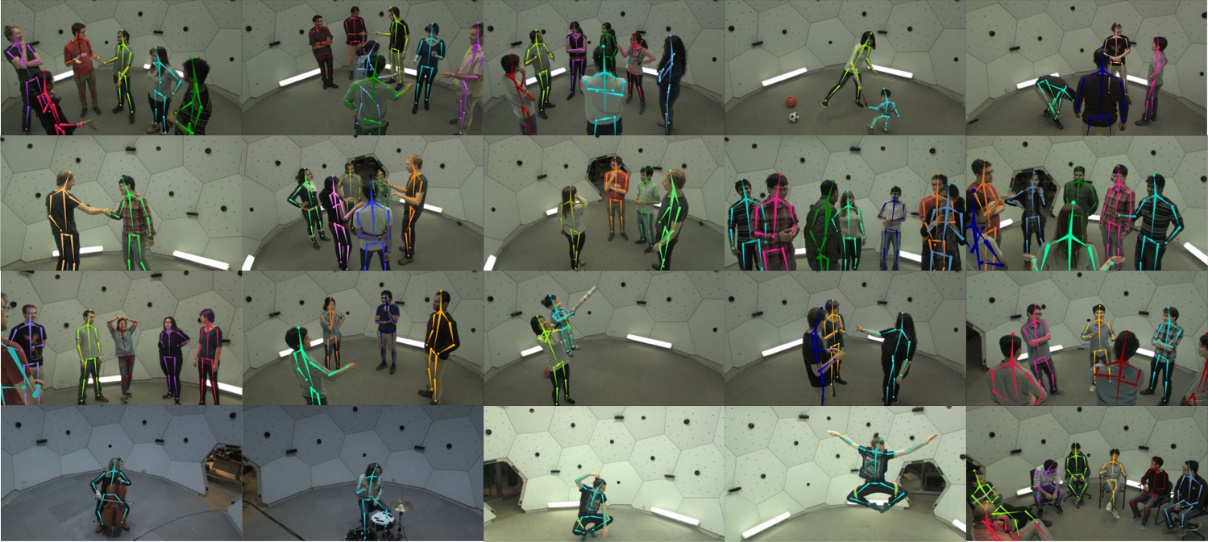


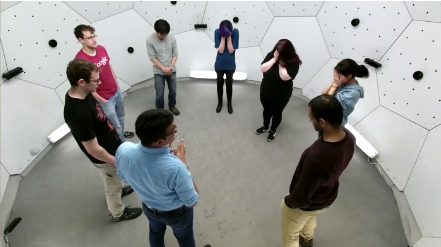

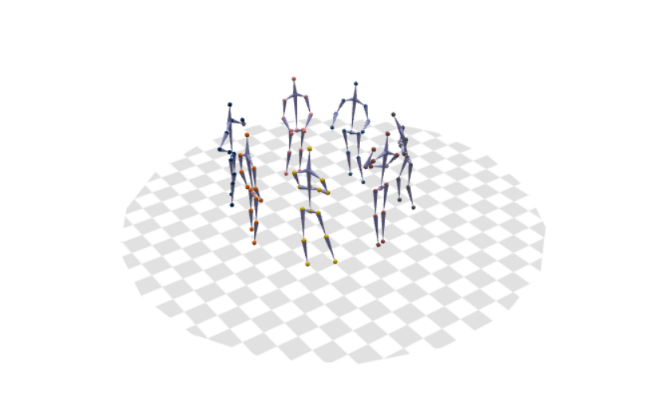


**S3 Fig.** Some example images of CMU Panoptic dataset and 3D ground truth joints

**Reference**

1. Belagiannis V, Amin S, Andriluka M, Schiele B, Navab N, Ilic S. 3D Pictorial Structures for Multiple Human Pose Estimation. Proceedings of the 2014 IEEE Conference on Computer Vision and Pattern Recognition (CVPR); 2014 Jun 23-28; Columbus, OH, USA: IEEE; 2014. p. 1669-1676.
2. Belagiannis V, Amin S, Andriluka M, Schiele B, Navab N, Ilic S. 3D Pictorial Structures Revisited: Multiple Human Pose Estimation. IEEE T. Pattern Anal. 2016;38(10):1929-1942.
3. Ershadi-Nasab S, Noury E, Kasaei S, Sanaei E. Multiple human 3D pose estimation from multiview images. *Multimed.* Tools Appl. 2018;77:15573-15601.
4. Joo H, Simon T, Li XL, Liu H, Tan L, Gui L, et al. Panoptic Studio: A Massively Multiview System for Social Interaction Capture. IEEE T. Pattern Anal. 2019;41(1):190-204.
5. Dong J, Jiang W, Huang Q, Bao H, Zhou X. Fast and Robust Multi-Person 3D Pose Estimation from Multiple Views. Proceedings of the 2019 IEEE/CVF Conference on Computer Vision and Pattern Recognition (CVPR); 2019 Jun 15-20; Long Beach, CA, USA: IEEE; 2019. p. 7792-7801.
